# AlkB homolog 3-mediated tRNA demethylation promotes protein synthesis in cancer cells

**DOI:** 10.1038/srep42271

**Published:** 2017-02-13

**Authors:** Yuko Ueda, Ikumi Ooshio, Yasuyuki Fusamae, Kaori Kitae, Megumi Kawaguchi, Kentaro Jingushi, Hiroaki Hase, Kazuo Harada, Kazumasa Hirata, Kazutake Tsujikawa

**Affiliations:** 1 Laboratory of Molecular and Cellular Physiology, Graduate School of Pharmaceutical Sciences, Osaka University, Osaka 565-0871, Japan; 2 Laboratory of Applied Environmental Biology, Graduate School of Pharmaceutical Sciences, Osaka University, Osaka 565-0871, Japan

## Abstract

The mammalian AlkB homolog (ALKBH) family of proteins possess a 2-oxoglutarate- and Fe(II)-dependent oxygenase domain. A similar domain in the *Escherichia coli* AlkB protein catalyzes the oxidative demethylation of 1-methyladenine (1-meA) and 3-methylcytosine (3-meC) in both DNA and RNA. AlkB homolog 3 (ALKBH3) was also shown to demethylate 1-meA and 3-meC (induced in single-stranded DNA and RNA by a methylating agent) to reverse the methylation damage and retain the integrity of the DNA/RNA. We previously reported the high expression of ALKBH3 in clinical tumor specimens and its involvement in tumor progression. In this study, we found that ALKBH3 effectively demethylated 1-meA and 3-meC within endogenously methylated RNA. Moreover, using highly purified recombinant ALKBH3, we identified N6-methyladenine (N6-meA) in mammalian transfer RNA (tRNA) as a novel ALKBH3 substrate. An *in vitro* translation assay showed that ALKBH3-demethylated tRNA significantly enhanced protein translation efficiency. In addition, ALKBH3 knockdown in human cancer cells impaired cellular proliferation and suppressed the nascent protein synthesis that is usually accompanied by accumulation of the methylated RNAs. Thus, our data highlight a novel role for ALKBH3 in tumor progression via RNA demethylation and subsequent protein synthesis promotion.

*Escherichia coli (E. coli*) AlkB protein is a 2-oxoglutarate (2-OG)- and Fe(II)-dependent oxygenase that catalyzes the direct oxidative demethylation of 1-meA and 3-meC and plays a central role in DNA and RNA methylation damage repair^1^,^2^,^3^. In humans, the AlkB homolog family consists of nine members (ALKBH1–ALKBH8 and fat mass and obesity-associated protein (FTO, also known as ALKBH9))^2^,^4^,^5^,^6^. We previously reported that ALKBH3 (originally named as prostate cancer antigen-1 (PCA-1)) is highly expressed in prostate cancer, and that knockdown of this homolog in prostate cancer cell lines significantly inhibits *in vitro* cell growth and *in vivo* tumor growth in a xenograft model^7^. In addition, a high level of ALKBH3 expression is also found in non–small-cell lung cancer^8^, pancreatic cancer^9^, and renal cell carcinoma^10^. Moreover, ALKBH3 overexpression is significantly linked to poor prognosis in cancer patients. These findings suggest that ALKBH3 may be a potential therapeutic target for some cancers.

Similar to AlkB, ALKBH3 expresses preferential demethylase activity for 1-meA and 3-meC in single-stranded DNA (ssDNA) over double-stranded DNA (dsDNA), whose methylation is induced by a methylating agent^1^,^5^. The methylation of ssDNA affects DNA replication by suppressing DNA base pairing through the hydrogen bonds. Therefore, the repair of ssDNA with methylation damage is essential for DNA replication and hence cell growth. The overexpression of ALKBH3 in some cancer cells is reported to repair the methylation damage in ssDNA, with the assistance of the helicase activating signal co-integrator complex 3 (ASCC3)^11^.

Accumulating evidence has established the association between DNA repair deficiencies and human diseases, including cancers, neurological diseases, and developmental defects^12^. On the other hand, the involvement of RNA damage repair in human diseases still remains obscure. Since ALKBH3 has a substrate preference for RNA as well as ssDNA, the defective repair of RNA methylation damage induced by endogenous and exogenous methylating agents may contribute to cancer progression. Among the ALKBH family members, ALKBH1, ALKBH 3, ALKBH5, and FTO have so far been reported to have RNA demethylase activity^13^,^14^,^15^,^16^. With regard to their subcellular localization, ALKBH1 and ALKBH3 are located in both the cytoplasm and nucleus^17^, whereas FTO is localized to the nucleus^6^ and ALKBH5 is reported to be in nuclear speckles^16^. Thus, these ALKBH family members may have different spatial and temporal functions in response to DNA/RNA methylation.

We report here that ALKBH3 expresses demethylation activity against 1-meA and 3-meC in RNA as well as N6-methyladenine (N6-meA) in only tRNA within endogenously methylated RNA bases. The demethylation of tRNA by recombinant ALKBH3 significantly enhances protein translation efficiency *in vitro*. Moreover, ALKBH3 knockdown increases the 1-meA level in RNA and decreases nascent protein levels in the PANC-1 cell line. These findings contribute to novel progress in the field of RNA epigenetics.

## Results

### ALKBH3 expresses demethylase activity for 3-meC, 1-meA, and N6-meA in RNA

**Figure 1:**
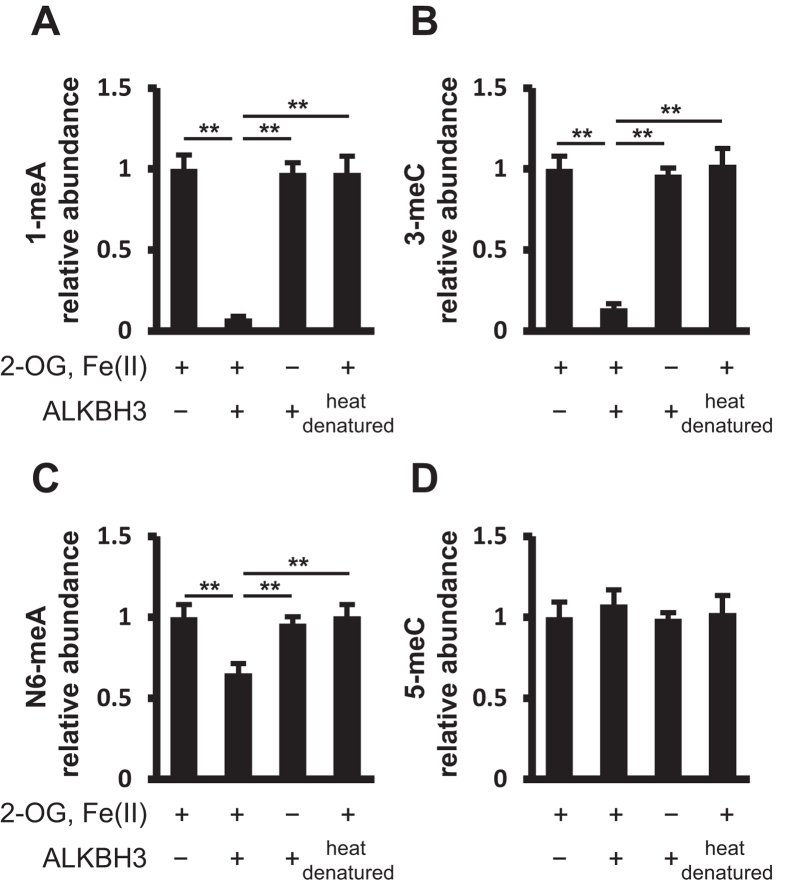
Recombinant ALKBH3 demethylates 3-meC, 1-meA, and N6-meA in total RNA. Total RNA purified from PANC-1 cells was incubated in the absence or presence of silkworm recombinant ALKBH3 and then enzymatically digested to nucleosides by treatment with nuclease P1, phosphodiesterase II, and alkaline phosphatase. The nucleosides were subjected to LC-ESI-MS/MS and the peak areas of methylated nucleosides were normalized for that of cytidine. Relative abundance levels of 1-meA (**A**), 3-meC (**B**), N6-meA (**C**), and 5-meC (**D**) are shown. ALKBH3 demethylated 3-meC, 1-meA, and N6-meA in the presence of 2-OG and Fe(II) but not in their absence. Heat-denatured ALKBH3 showed no enzymatic activity for the methylated bases. ALKBH3 did not demethylate 5-meC, even in the presence of 2-OG and Fe(II). Data show means ± S.D. (n = 3). **p < 0.01.

Various RNA bases are known to be methylated, including 1-meA, 3-meC, 5-methylcytosine (5-meC), and N6-meA. To evaluate the RNA demethylation activity of ALKBH3, total RNA purified from the human pancreatic cancer cell line PANC-1 was incubated with the silkworm-derived recombinant ALKBH3 protein^18^. The recombinant ALKBH3 is confirmed to have higher demethylation activity for 3-meC in the synthesized DNA oligonucleotide than commercially available *E. coli* recombinant ALKBH3. First, we focused on 1-meA, 3-meC, N6-meA, and 5-meC. The reason is that the methylating agent-induced 1-meA and 3-meC in DNA/RNA are elucidated to be demethylated by ALKBH3, and N6-meA in mRNA was recently clarified to be a substrate for ALKBH5 and FTO (ALKBH9)^15^,^16^. As determined by LC-ESI-MS/MS, ALKBH3 completely eliminated 1-meA and 3-meC in the total RNA, suggesting that these two endogenously methylated RNA bases are also substrates for ALKBH3 (Fig. 1A and B, Supplementary Fig. S1). In addition, we found that ALKBH3 could demethylate N6-meA, although the demethylation ratio was approximately half of that for 1-meA and 3-meC (Fig. 1C). The enzymatic activity of ALKBH3 had an absolute requirement for 2-OG and Fe(II), as reported in the experimental setting of AlkB^1^. Heat-denatured silkworm-derived recombinant ALKBH3 show no demethylation activity for those methylated bases. As expected, we observed no RNA demethylation activity for 5-meC (Fig. 1D). Thus, ALKBH3 expresses demethylase activity for 1-meA, 3-meC, and N6-meA in total RNA.

### ALKBH3 preferentially demethylates N6-meA in tRNA

**Figure 2:**
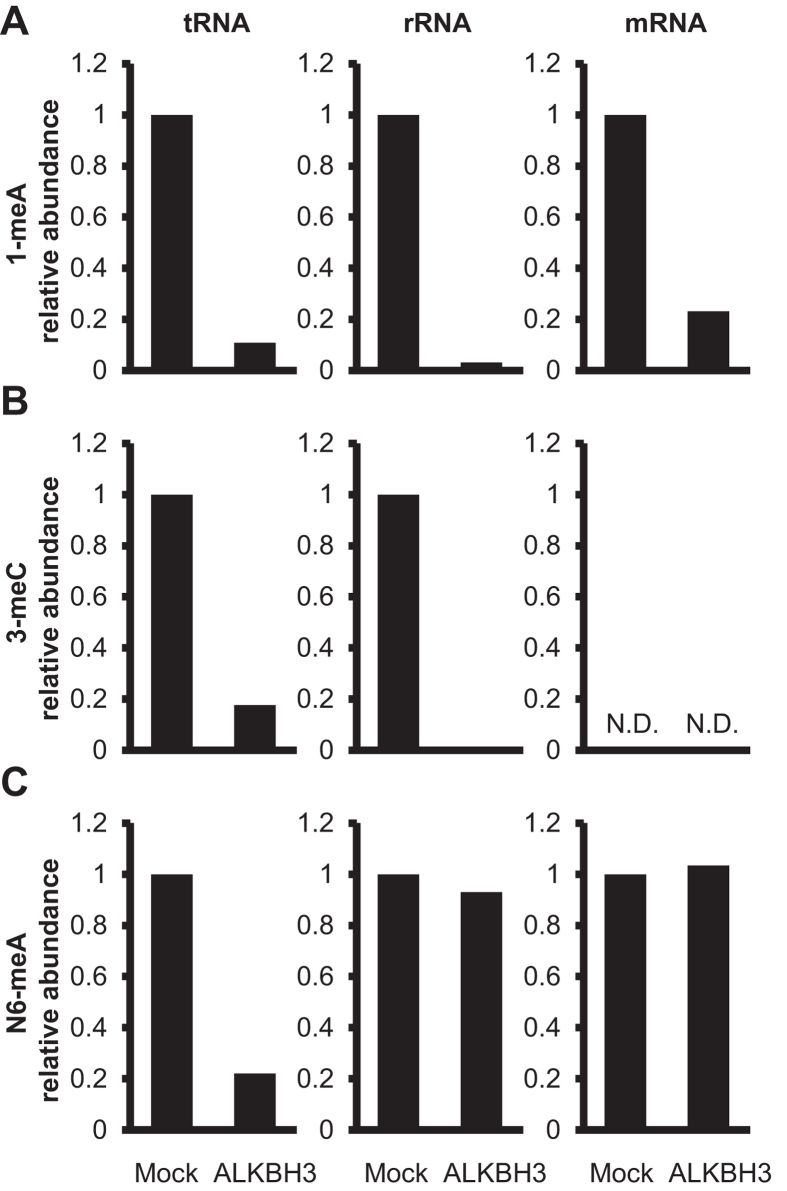
ALKBH3 has substrate preference for N6-meA in tRNA. Bovine tRNA, rRNA, and mRNA were incubated in the absence (Mock) or presence of ALKBH3, enzymatically degraded to nucleosides, and then analyzed the methylation status of 1-meA (**A**), 3-meC (**B**), and N6-meA (**C**) by LC-ESI-MS/MS. The peak areas of methylated nucleosides were normalized to that of cytidine. ALKBH3 demethylated only N6-meA in tRNA, 3-mC in tRNA and rRNA, and 1-mA in the three kinds of RNAs. The peak of 3-meC in mRNA was not sufficient for a quantitative determination (N.D.: not determined).

To further investigate the substrate preference of ALKBH3, we incubated tRNA, messenger RNA (mRNA), and ribosomal RNA (rRNA) with the recombinant ALKBH3 and analyzed the methylation status of 1-meA, 3-meC, and N6-meA by LC-ESI-MS/MS. The recombinant ALKBH3 almost completely demethylated 1-meA in all three types of RNAs (Fig. 2A). On the other hand, demethylation of 3-meC was detectable in rRNA and tRNA, but not in mRNA, in which basal 3-meC was under the detectable level (Fig. 2B). Interestingly, N6-meA was found to be demethylated only in tRNA, but not in rRNA or mRNA (Fig. 2C), suggesting that ALKBH3 had a unique substrate preference for methylated RNA bases.

**Figure 3:**
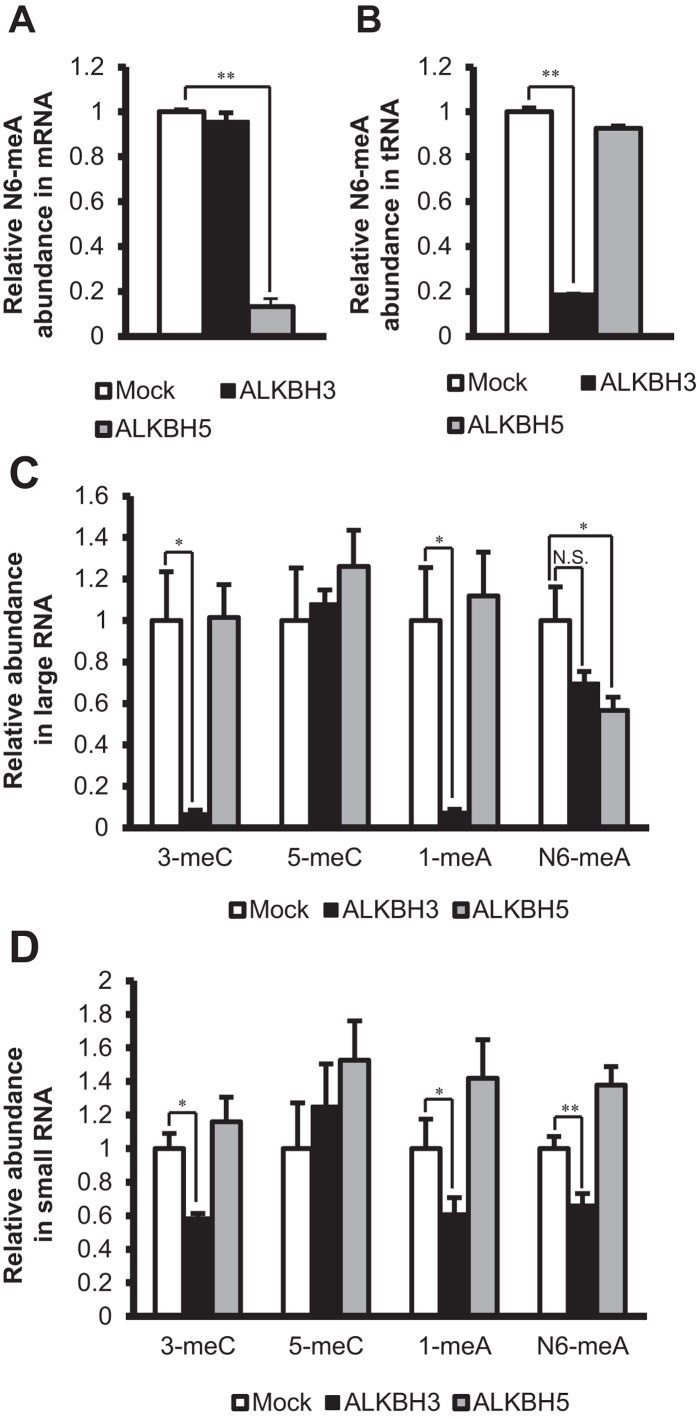
ALKBH3 and ALKBH5 have distinct roles in RNA demethylation. The larger RNA fraction, smaller RNA fraction, tRNA, and mRNA were incubated in the absence (Mock) or presence of ALKBH3 or ALKBH5, enzymatically degraded to nucleosides, and then subjected to LC-ESI-MS/MS. The peak areas of methylated nucleosides were normalized to that of cytidine. (**A**) ALKBH5, but not ALKBH3, demethylated N6-meA in mRNA. (**B**) ALKBH3, but not ALKBH5, demethylated N6-meA in tRNA. (**C**) In the larger RNA fraction, ALKBH3 demethylated 3-meC and 1-meA. On the other hand, ALKBH5 demethylated only N6-meA. (**D**) In the smaller RNA fraction, ALKBH3 demethylated 3-meC, 1-meA, and N6-meA. On the other hand, ALKBH5 did not demethylate any of the methylated nucleosides. Data show means ± S.D. (n = 3). N.S.: *p* > 0.05, *p < 0.05, **p < 0.01.

Since ALKBH5 was recently reported to exhibit demethylation activity for N6-meA in mRNA, we compared the demethylation activities of recombinant ALKBH3 and ALKBH5 proteins purified from silkworm pupae (Supplementary Fig. S2), using commercially available bovine mRNA and tRNA as substrates. As shown in Fig. 3A, the demethylation activity of ALKBH5 for N6-meA in mRNA was confirmed, whereas ALKBH3 had no such activity. On the other hand, ALKBH3 showed marked demethylation activity for N6-meA in tRNA, but ALKBH5 did not (Fig. 3B).

To confirm the demethylation specificity of ALKBH3 in human RNAs, we size-fractionated the RNAs of PANC-1 cells into two fractions containing RNAs larger and smaller than approximately 200 nucleotides in length, respectively (Supplementary Fig. S3). The larger and the smaller RNA fractions mainly contained mRNA and rRNA, and tRNA and microRNA (miRNA), respectively. ALKBH3 demethylated 3-meC and 1-meA in the larger RNA fraction (Fig. 3C). On the other hand, ALKBH5 expressed demethylase activity only for N6-meA, supporting the reported finding of ALKBH5-mediated N6-meA demethylation in mRNA. Interestingly, ALKBH3 demethylated not only 1-meA and 3-meC but also N6-meA in the smaller RNA fraction containing tRNA (Fig. 3D). However, ALKBH5 did not exhibit any demethylase activity for the methylated RNA bases in this smaller fraction. These results indicate that ALKBH3 and ALKBH5 recognize N6-meA in combination with the higher-order structure of the RNA.

### ALKBH3-demethylated tRNA enhances *in vitro* protein translation efficiency

**Figure 4:**
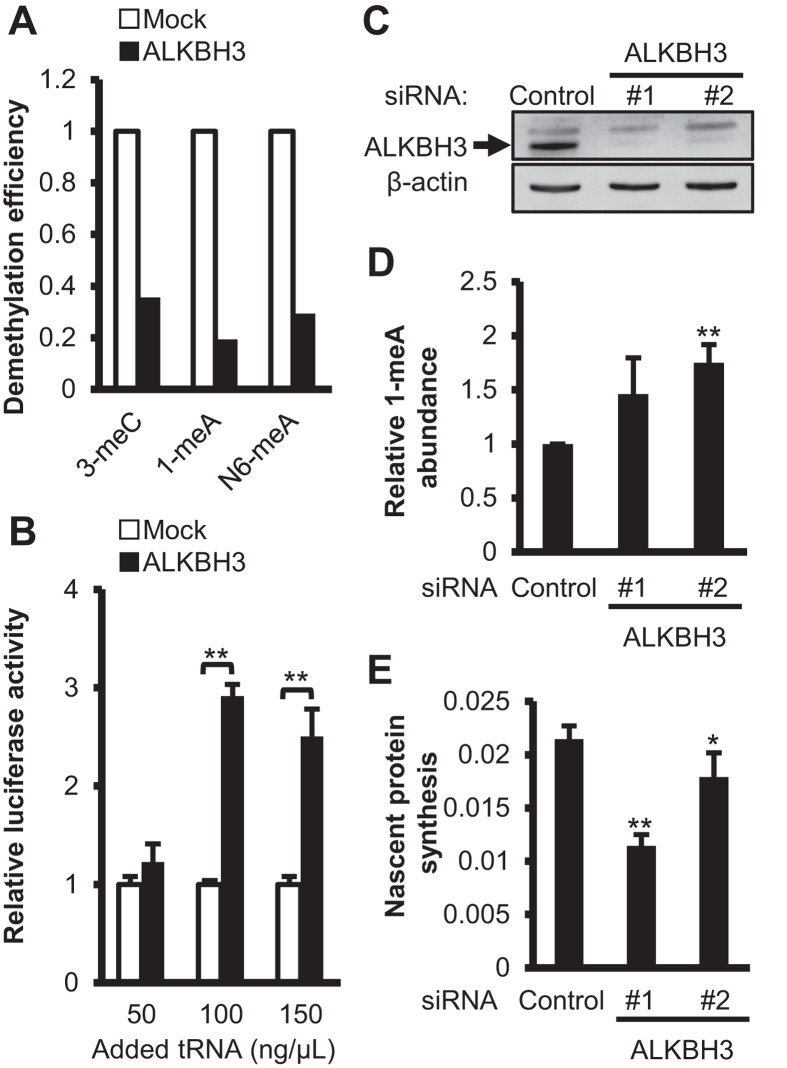
ALKBH3-pretreated tRNA showed increased *in vitro* translation efficiency and ALKBH3 knockdown decreased nascent protein synthesis of pancreatic cancer cells accompanying accumulation of the methylated RNA. (**A**) tRNA was incubated in the absence (Mock) or presence of ALKBH3 for 2 h and used for *in vitro* translation. The demethylation efficiency of tRNA was determined by LC-ESI-MS/MS analysis. Data show means ± S.D. (n = 3). **: *P* <  0.01. (**B**) The *in vitro* translation mixture was incubated for 10 min at 30 °C and the resultant luciferase activity was measured. (**C**) Immunoblotting confirmed the successful knockdown of ALKBH3 expression in the transfected PANC-1 cells. The full length western blot are presented in Supplementary Fig. 4S. (**D**) Detection of 1-meA levels in the smaller RNA fractions from control and ALKBH3 siRNA-transfected PANC-1 cells. After extraction and purification, serial dilutions of the smaller RNA fraction (200, 100, 50, and 25 ng) were blotted and probed using an anti-1-meA antibody. Data show means ± S.D. (n = 3). **p < 0.01. (**E**) Nascent protein synthesis in PANC-1 cells transfected with control or ALKBH3 siRNAs was assessed using the Click-iT AHA Alexa Fluor 488 Protein Synthesis HCS Assay. Data show means ± S.D. (n = 5). *p < 0.05, **p < 0.01.

The difference between ALKBH3 and ALKBH5 in their preference of substrate in the smaller RNA fraction prompted us to examine the effect of ALKBH3 on protein translation through tRNA demethylation. We incubated bovine tRNA with the recombinant ALKBH3 protein or the mock control in the presence of 2-OG and Fe(II), and confirmed the demethylation of 1-meA, 3-meC, and N6-meA by LC-ESI-MS/MS (Fig. 4A). Subsequently, the ALKBH3- and mock control-treated tRNAs were subjected to the *in vitro* translation system of luciferase mRNA using rabbit reticulocyte lysate, and the activity of the translated luciferase protein was measured in the incubation mixture by a luminometer. As shown in Fig. 4B tRNA preincubated with the recombinant ALKBH3 induced significantly higher luciferase activity than that with the mock control, indicating that ALKBH3-mediated tRNA demethylation led to increased protein translation efficiency.

### ALKBH3 knockdown in cancer cells decreases protein synthesis associated with methylated RNA accumulation

Finally, to investigate the effect of ALKBH3-mediated RNA demethylation on protein synthesis in cancer cells, we knocked down ALKBH3 by using siRNAs that target different sequences of the human *ALKBH3* gene in the PANC-1 cell line (which has high expression of ALKBH3)^9^. Immunoblot and real-time PCR analyses confirmed the successful knockdown of ALKBH3 protein and mRNA, respectively, in transfected PANC-1 cells (Fig. 4C and Supplementary Fig. S5). The ALKBH3-knockdown PANC-1 cells displayed a substantial accumulation of 1-meA in the smaller RNA fraction, as demonstrated by dot blot quantification using an anti-1-meA antibody (Fig. 4D). We also attempted to determine the N6-meA levels in the same amount of RNA used for 1-meA quantification by a dot blot analysis with a commercially available anti-N6-meA antibody. However, we could not detect N6-meA in the smaller RNA fractions. The absolute amount of N6-meA was much less than that of 1-meA in the fraction containing tRNA. In addition, immunocytochemical examination detected abundant 1-meA accumulation, especially in the cytoplasm, of the ALKBH3-knockdown PANC-1 cells compared with the control siRNA-treated cells (Supplementary Fig. S6). To evaluate whether the ALKBH3 knockdown affects cellular protein translation, we determined the rate of nascent protein synthesis by measuring the incorporation of methionine analogs. As shown in Fig. 4E, ALKBH3 knockdown significantly decreased the nascent protein synthesis in the PANC-1 cells. These results suggest that overexpression of ALKBH3 enhances protein translation efficiency through tRNA demethylation, which is essential for active growth of cancer cells. In agreement with these findings, ALKBH3 knockdown reduced the growth of PANC-1 cells (Supplementary Fig. S7).

## Discussion

Over 100 structurally distinct RNA modifications have been identified so far^19^. These post-transcriptional modifications are widely present in various RNAs, including tRNA, mRNA, rRNA, miRNA, long non-coding RNA, etc. However, the enzyme regulatory mechanisms as well as the biological significance of RNA modification are not yet fully understood and are attracting the most attention in current RNA research. We report here that ALKBH3 is a demethylase not only for 1-meA and 3-meC in mammalian RNAs but also for N6-meA in tRNA, and that tRNA demethylation by ALKBH3 increases protein translation efficiency.

The function of ALKBH3 has been mainly studied in the context of DNA demethylation. ALKBH3 preferentially demethylates 1-meA and 3-meC in ssDNA over dsDNA that are induced by methylating agents such as methyl methanesulfonate, to repair harmful DNA methylation damage. Recently, it was elegantly shown that ALKBH3 is associated with the helicase ASCC3 to demethylate endogenously methylated 3-meC in ssDNA in prostate cancer cells^11^. Moreover, ALKBH3 seems to bind to the transcription initiation sites of some highly active gene promoters^20^. Therefore, highly expressed ALKBH3 would maintain genome integrity through these regulation mechanisms in actively proliferating cancer cells.

In RNA, repair functions of ALKBH3 on methylated lesions have also been proposed, ALKBH3 can demethylate 1-meA and 3-meC in an RNA oligonucleotide, reactivates bacteriophage ssRNA that had been treated by methylating agents^1^,^21^. Subsequently, Ougland *et al*. demonstrated that the luciferase mRNA and *E. coli* tRNA exposed to methylating agents were repaired by ALKBH3 to recover their functions^13^. Based on these results, our novel finding in the present study using mammalian naturally occurring cellular RNAs is that ALKBH3 has demethylation activity for the endogenously methylated RNA bases 1-meA and 3-meC as well as N6-meA in tRNA. N6-meA in tRNA, but not in rRNA or mRNA, was demethylated by ALKBH3, suggesting that ALKBH3 had a unique substrate preference for methylated RNA types. tRNA has a unique three-dimensional conformation called the cloverleaf structure, which prompted us to examine the role of this conformation in N6-meA demethylation by ALKBH3. Accordingly, heat-denatured and undenatured tRNAs were incubated with recombinant ALKBH3. We did not detect a significant difference in N6-meA demethylation between these two groups (Supplementary Fig. S8). Further studies are needed to clarify the substrate preference of ALKBH3 for various RNA types.

Evidence is accumulating with regard to the location and function of N6-meA, which N6-meA is the most abundant internal modification in poly(A)^+^ RNA, including mRNA, and is relatively abundantly located near the stop codons and in long internal exons as well as in the 3′UTR of mRNA^22^,^23^. Among the biological importance of N6-meA are its involvement in mRNA stability^24^, translation-elongation dynamics^25^, and pri-miRNA processing^26^. The dynamic processes regulated by N6-meA modification modulate cellular functions such as the speed of the circadian clock^27^,^28^ and self-renewal capability of embryonic stem cells and somatic cell reprogramming^29^,^30^.

Recently, FTO^15^ and ALKBH5^16^ were reported to have demethylation activity for N6-meA in mRNA. However, their demethylation activity for N6-meA in tRNA has not been examined. Our present study shows that ALKBH3, but not ALKBH5, possesses demethylase activity for N6-meA in tRNA and that N6-meA in mRNA is a substrate for ALKBH5, but not for ALKBH3. The regulation of methylation is based on the fine tuning between methylase and demethylase. Some tRNA methyltransferases (e.g., DNA methyltransferase, NOP2/Sun RNA methyltransferase family member 2, tRNA methyltransferase 2 homolog A, ALKBH8, etc.) have been identified in mammals^31^. However, no mammalian tRNA demethylase has been reported so far. To our best knowledge, ALKBH3 is the first mammalian tRNA demethylase to have been identified, which has substrate specificity for at least N6-meA, 1-meA, and 3-meC.

Other important findings in this study are that the efficiency of *in vitro* translation was increased by addition of ALKBH3-treated tRNA compared with control tRNA, and that knockdown of ALKBH3 in PANC-1 cancer cells significantly suppressed the translation of nascent protein accompanying the accumulation of 1-meA in the tRNA-enriched smaller RNA fraction. These results indicate that the demethylation of tRNA catalyzed by ALKBH3 would affect protein translation efficiency in cells. Although the mechanism remains unclear, aminoacylation of bacterial tRNA treated with the methylating agent dimethyl sulfate was reported to be inhibited^13^, suggesting that the enzymatic activity of ALKBH3 may contribute to aminoacyl-tRNA synthesis. Global control of protein synthesis is a crucial component of cancer development and progression^32^, as highly proliferating cancer cells require increased protein synthesis^33^. Related to these findings, ALKBH3-knockdown PANC-1 cells exhibited decreased cell proliferation. Therefore, the increased protein synthesis mediated by ALKBH3 demethylase possibly leads to the promotion of tumor development and progression *in vivo*.

In summary, our findings reveal that ALKBH3 demethylates endogenously methylated 1-meA and 3-meC in RNA as well as N6-meA in tRNA and that ALKBH3-modified tRNA increases protein translation efficiency. These results represent new findings in RNA epigenetics. Moreover, although further investigation is required to understand the molecular relationship between methylated tRNA bases and the translation machinery, ALKBH3 demethylase would be a promising therapeutic target for cancer with a novel action mechanism of regulating RNA methylation.

## Materials and Methods

### Reagents and cell culture

Bovine liver tRNA and calf liver rRNA were purchased from Sigma-Aldrich (St. Louis, MO, USA). Bovine kidney mRNA was purchased from Clontech Laboratories, Inc. (Mountain View, CA, USA). The human pancreatic cancer cell line PANC-1 was obtained from the American Type Culture Collection (ATCC, Manassas, VA, USA) and was grown at 37 °C under 5% CO_2_ atmosphere in RPMI 1640 medium (Wako Pure Chemical Industries, Osaka, Japan) supplemented with 10% heat-inactivated fetal calf serum and 100 μg/ml kanamycin.

### RNA isolation and confirmation of purified RNAs

Total RNA and a larger RNA fraction (excluding RNAs of less than 200 nucleotides in length) were isolated from the PANC-1 cells with the miRNeasy Mini Kit (QIAGEN Inc., Valencia, CA, USA) according to the manufacturer’s protocol. A smaller RNA fraction (enriched RNAs of less than 200 nucleotides in length) was isolated from the PANC-1 cells with the miRNeasy Mini Kit and RNeasy MinElute Cleanup Kit (QIAGEN) according to the manufacturer’s protocol. The purified RNA was analyzed with an Experion automated electrophoresis system using the RNA StdSens Analysis Kit (Bio-Rad Laboratories Inc., Hercules, CA, USA).

### ALKBH3-mediated RNA demethylation

Recombinant FLAG-His-tagged ALKBH3 was expressed in silkworm pupae and purified by column chromatography as described previously^18^. RNAs were incubated for 2 h at 37 °C with the recombinant ALKBH3 in 50 mM Tris-HCl (pH 8.0), 2 mM ascorbate, 0.1 mM 2-OG, and 40 μM FeSO_4_. Subsequently, the RNAs were purified by ethanol precipitation for further examination.

### LC-ESI-MS/MS analysis of nucleosides from RNA

Five microliters of 0.1 M ammonium acetate (pH 5.3) and 0.5 units of nuclease P1 (Wako) were added to the purified RNA sample (in 45 μl of H_2_O) and then incubated for 2 h at 45 °C. Subsequently, 5.5 μl of 1 M ammonium bicarbonate and 0.002 units of venom phosphodiesterase II (Wako) were added to the solution. The incubation was continued for an additional 2 h at 25 °C. Thereafter, the mixture was incubated for 1 h at 37 °C with 0.5 units of alkaline phosphatase (New England BioLabs, Ipswich, MA, USA). Then, 1.3 μl of 0.1 N HCl, 50 μl of H_2_O, and 20 μl of chloroform were added to the mixture. The sample was vortexed and the resulting suspension was centrifuged for 5 min at 5,000 *g*. The aqueous layer was collected and evaporated to dryness. The resulting nucleoside residues were solubilized in Milli-Q water (Merck Millipore, Billerica, MA, USA). Liquid chromatography-electrospray ionization-tandem mass spectrometry (LC-ESI-MS/MS) analyses were performed on a Waters ACQUITY UPLC system (Waters Corp., Milford, MA, USA) coupled to a Quattro Premier XE triple quadrupole mass spectrometer (Waters). LC separations were carried out on a COSMOSIL Cholester 2.5 μm, 2.0 × 100 mm column (Nacalai Tesque, Kyoto, Japan) at 50 °C, at a flow rate of 0.3 ml/min. The mobile phase consisted of solvent A (5 mM ammonium formate and 0.2% (v/v) formic acid) and solvent B (acetonitrile), starting with 100% solvent A/0% solvent B for 2 min, followed by a 3-min linear gradient of 0 to 10% solvent B, a 1-min linear gradient of 10% to 90% solvent B, 4 min with 90% solvent B, and 5 min re-equilibration with the initial mobile phase conditions. The data at one run were acquired for 15 min. The mass spectrometer was operated using an ESI source in the positive mode. ESI-MS/MS was conducted in the negative-ion mode. The ionization parameters were capillary voltage, 3.0 kV; extractor voltage, 2 V; source temperature, 120 °C; desolvation temperature, 350 °C; desolvation gas flow, 800l/h; and cone gas flow, 50l/h. The selected reaction monitoring (SRM) transitions (*m/z* of precursor ion/*m/z* of product ion) and parameters (cone voltage and collision energy) for nucleosides are listed in Supplementary Table S1. The interchannel delay and interscan delay were set at 0.01 s and 0.05 s, respectively. The dwell time for each SRM was set at 50 ms.

### *In vitro* translation reaction and luciferase assay

The Flexi Rabbit Reticulocyte Lysate System (Promega, Madison, WI, USA) was used. The reaction was performed for 10 min at 30 °C in a 10 μl reaction mixture, containing 7.0 μl of Flexi rabbit reticulocyte lysate, 0.1 μl of 1 mM amino acid mixture without leucine, 0.1 μl of 1 mM amino acid mixture without methionine, 0.28 μl of 2.5 M potassium chloride, 0.2 μg of luciferase control RNA, and silkworm recombinant ALKBH3- or mock control-treated bovine tRNA. Subsequently, the luciferase activity was measured on a 20/20n luminometer (Promega) using a luciferase assay system (Promega) according to the manufacturer’s protocol.

### ALKBH3 knockdown by siRNA transfection and western blot analysis

PANC-1 cells (1 × 10^5^) were seeded in a 6-well plate and transfected either with 25 nM control siRNA or ALKBH3 siRNA (B-Bridge International, Inc., Santa Clara, CA, USA) using the Lipofectamine 2000 Reagent (Invitrogen, Carlsbad, CA, USA) according to the manufacturer’s protocol. The control and ALKBH3 siRNA sequences were as follows: 5′-auccgcgcgauaguacgua-3′ and 5′-uacguacuaucgcgcggau-3′ for control siRNA; 5′-gagagaagcuucacugaaa-3′ and 5′-uuucagugaagcuucucuc-3′ for ALKBH3 siRNA#1; and 5′-gaaagaagcugacuggaua-3′ and 5′-uauccagucagcuucuuuc-3′ for ALKBH3 siRNA#2. At 48 h after transfection, the cells were harvested. Cell lysates were resolved on a 10% sodium dodecyl sulfate (SDS)-polyacrylamide gel (Bio-Rad) and transferred to a polyvinylidene difluoride membrane (PVDF; Merck Millipore). The membranes were blocked with 3% bovine serum albumin at room temperature for 1 h. Thereafter, the membranes were incubated overnight with anti-ALKBH3 antibody (09–882; Merck Millipore) at 4 °C or for 1 h with anti-β-actin antibody (Sigma-Aldrich) at room temperature. This was followed by incubation with horseradish peroxidase (HRP)-conjugated anti-rabbit IgG or anti-mouse IgG (Santa Cruz Biotechnology, Dallas, TX, USA) for 1 h. Bound HRP conjugates were visualized using the enhanced chemiluminescence reagent (GE Healthcare, Pittsburgh, PA, USA) and captured with the ImageQuant LAS 4000 imager (GE Healthcare).

### Dot blot analysis

At 48 h after ALKBH3 siRNA transfection, a smaller RNA fraction was purified using the miRNeasy Mini Kit (QIAGEN) and RNeasy MinElute Cleanup Kit (QIAGEN) according to the manufacturer’s protocols. The purified RNA samples were denatured at 95 °C for 5 min and then immediately chilled on ice. Two-fold serial dilutions of the denatured RNA samples were spotted on a PVDF membrane. The membrane was washed with 2× saline sodium citrate buffer, air-dried, and baked at 80 °C for 2 h. The membrane was blocked with 5% skim milk in phosphate-buffered saline for 1 h at room temperature and then incubated overnight with anti-N1-methyladenosine antibody (D-345–3; MBL, Nagoya, Japan) at 4 °C, followed by incubation with HRP-conjugated anti-mouse IgG antibody. Bound HRP conjugates were visualized using the enhanced chemiluminescence reagent (GE Healthcare).

### Quantification of nascent protein synthesis

Nascent protein synthesis was measured *in vitro* using the Click-iT AHA Alexa Fluor 488 Protein Synthesis HCS Assay (Invitrogen; Life Technologies, Carlsbad, CA, USA). At 48 h after transfection of cells with ALKBH3 siRNA, the culture medium was replaced with methionine-free RPMI 1640 medium (Invitrogen; Life Technologies) containing 50 μM of the methionine analog l-azidohomoalanine (AHA) and the cells were incubated for 2 h. The medium was then replaced to remove free AHA. The cells were fixed with 3.7% formaldehyde for 15 min and permeabilized with 0.5% Triton X-100 for 20 min at room temperature. The AHA was stained using a Click-iT reaction cocktail (with fluorescein isothiocyanate (FITC)), and the nuclei were stained using Hoechst 33342 dye. Fluorescence measurement was carried out by using the Envision Multilabel Reader (PerkinElmer, Waltham, MA, USA) with the following filters: for Hoechst 33342, excitation/emission = 340/440 nm; and for FITC-Alexa Fluor 488, excitation/emission = 485/535 nm. The nascent protein synthesis ability was calculated from the fluorescence intensities of FITC-Alexa Fluor 488/Hoechst 33342.

### Statistical Analysis

Statistically significant differences between the control group and the treated group were determined using the unpaired Student’s *t*-test.

## Additional Information

**How to cite this article**: Ueda, Y. *et al*. AlkB homolog 3-mediated tRNA demethylation promotes protein synthesis in cancer cells. *Sci. Rep.* **7**, 42271; doi: 10.1038/srep42271 (2017).

**Publisher's note:** Springer Nature remains neutral with regard to jurisdictional claims in published maps and institutional affiliations.

## Supplementary Material

Supplementary Information
