# Supplementary material for: AlkB homolog 3-mediated tRNA demethylation promotes protein synthesis in cancer cells
Source: Sci Rep. 2017 Feb 13;7:42271. doi: 10.1038/srep42271 (PMC5304225; doi:10.1038/srep42271)

Supplementary Information

AlkB homolog 3-mediated tRNA demethylation promotes protein synthesis in cancer cells

Yuko Ueda[1], Ikumi Ooshio[1], Yasuyuki Fusamae[1], Kaori Kitae[1], Megumi Kawaguchi[1], Kentaro Jingushi[1], Hiroaki Hase[1], Kazuo Harada[2], Kazumasa Hirata[2] and Kazutake Tsujikawa[1]*

Supplementary Materials and Methods

Immunoblot analysis of recombinant ALKBH3 and ALKBH5 from silkworm

Recombinant ALKBH3 and ALKBH5 from silkworm pupae were resolved on a 10% SDS-polyacrylamide gel (Bio-Rad) and transferred to a PVDF membrane (Millipore). The membrane was blocked with 3% bovine serum albumin for 1 h at room temperature. The membrane was then incubated overnight with anti-ALKBH3 antibody (Millipore; 09-882) or anti-ALKBH5 antibody (generated by our laboratory) at 4°C and then incubated with HRP-conjugated anti-rabbit IgG (Santa Cruz Biotechnology) at room temperature for 1 h. Bound HRP conjugates were visualized using the enhanced chemiluminescence reagent (GE Healthcare) and captured with the ImageQuant LAS 4000 imager (GE Healthcare).

Quantitative real-time PCR

Total RNA was extracted from cells using the miRNeasy Mini Kit (QIAGEN) according to the manufacturer’s protocol and cDNA was synthesized using a PrimeScript RT Reagent Kit (Takara Bio Inc., Shiga, Japan). Relative *ALKBH3* mRNA expression was quantified by real-time PCR using SYBR Premix Ex Taq (Takara). The following primers were used for the RT-PCR amplification of *ALKBH3*: *ALKBH3* forward, 5′-TAC CAC TGC TAA GAG CCA TCT CC-3′ and reverse 5′-GAC AGG CTG ATT TCA TAC ACA CC-3′. The PCR results were normalized using the quantity of glyceraldehyde-3-phosphate dehydrogenase (*GAPDH*) transcripts, which were determined with the following primer sequences: forward 5′-CCA TCA CCA TCT TCC AGG AG-3′ and reverse 5′-AAT GAG CCC CAG CCT TCT CC-3′.

Immunofluorescence staining

PANC-1 cells were transfected with control siRNA or ALKBH3 siRNA on a 12-well plate containing coverslips. After 48 h of transfection, the cells on the coverslips were fixed with 4% formaldehyde for 15 min and then permeabilized with 0.5% Triton X-100 for 20 min. After blocking

with 5% skim milk at room temperature for 1 h, the cells were incubated with anti-N1-methyladenosine antibody (1:500 dilution; MBL) for 1 h at room temperature and subsequently incubated with Alexa Fluor 488-conjugated goat anti-mouse IgG (1:1,000 dilution; Life Technologies) for 1 h at room temperature. Cell nuclei were stained with 4′,6-diamidino-2′-phenylindole dihydrochloride (DAPI). Stained cell images were captured by a BX2N-FL-2 fluorescence microscope (Olympus, Tokyo, Japan).

Cell proliferation assay

Cell proliferation was assessed using WST-1 reagent (Dojindo Molecular Technologies, Kumamoto, Japan) according to the manufacturer's protocol. At 48 h after transfection with control siRNA or ALKBH3 siRNA, the cells were replated on 96-well microplates. WST-1 reagent with 10% 1-methoxy-5-methylphenazinium methyl sulfate (Dojindo) was added to each well and the absorbance was measured after 2 h using the model 680 microplate reader (Bio-Rad) with a test wavelength of 450 nm and a reference wavelength of 630 nm.

Supplementary Figure Legends

Supplementary Figure S1. Mass chromatograms for nucleosides analysis of demethylation by ALKBH3.

(**A**) Mass chromatograms of 1-meA and N6-meA. (**B**) Mass chromatograms of 3-meA and 5-meA. The top, middle, and lower panels show the relative abundance levels of methylated nucleosides in RNAs incubated with 2-OG and Fe(II) without ALKBH3, ALKBH3 with 2-OG and Fe(II), and ALKBH3 without 2-OG and Fe(II), respectively.

Supplementary Figure S2. Immunoblot analysis of recombinant ALKBH3 and ALKBH5

Immunoblot analysis of purified silkworm recombinant FLAG-His-ALKBH3 protein (1 ng) using anti-ALKBH3 antibody (**A**) and silkworm recombinant FLAG-His-ALKBH5 protein (2.5 ng) using

anti-ALKBH5 antibody (**B**).

Supplementary Figure S3. Electropherogram of larger and smaller RNA fractions.

Each RNA fraction was analyzed using Experion (Bio-Rad) according to the manufacturer's protocol. (**A**) Digital gel image of the electropherogram. (**B**) The larger RNA fraction contained two major peaks corresponding to 18S and 28S ribosomal RNAs. (**C**) One major peak derived from tRNA and smaller-sized RNAs (<200 nucleotides) was observed in the smaller RNA fraction.

Supplementary Figure S4. Full length western blot of Fig. 4 (c).

The full length western blots of ALKBH3 and β-actin used in Fig. 4 (c) are presented.

Supplementary Figure S5. *ALKBH3* mRNA knockdown efficiency in PANC-1 cells.

The knockdown efficiency of *ALKBH3* mRNA using siRNAs was determined by real-time PCR. *ALKBH3* mRNA expression levels in ALKBH3 siRNA-transfected PANC-1 cells were reduced to approximately 20% of those of control siRNA-transfected cells. Data are presented as means ± S.D. (n = 3). **: $p < 0.01$.

Supplementary Figure S6. Immunofluorescence staining of 1-methyladenine in ALKBH3-knockdown PANC-1 cells.

Accumulation of 1-meA was detected in ALKBH3-knockdown PANC-1 cells using anti-1-meA antibody followed by Alexa 488-conjugated anti-mouse IgG (green). DAPI was used for nuclear staining (blue). Top panels show control siRNA-transfected PANC-1 cells. Middle and lower panels show ALKBH3 siRNA-transfected PANC-1 cells. Scale bars indicate 50 μm.

Supplementary Figure S7. Reduced cell growth of PANC-1 cells by ALKBH3 knockdown.

At 48 h after transfection with control siRNA or two ALKBH3 siRNAs (#1 and #2), PANC-1 cells were replated on 96-well plates for the growth analysis. The cell growth was measured by WST-1

assay and expressed as relative cell growth.

Supplementary Figure S8. Relative N6-meA levels in heat-denatured tRNA treated with ALKBH3.

Bovine tRNA was denatured at 70°C for 15 min and then immediately chilled on ice. Denatured and undenatured bovine tRNA were incubated in the absence (Mock) or presence of recombinant ALKBH3, enzymatically degraded to nucleosides, and then subjected to LC-ESI-MS/MS. The peak areas of methylated nucleosides were normalized to those of cytidine. There was no significant difference between these two groups. Data are presented as means ± S.D. (n = 3). *: $p < 0.05$, N.S.: $p > 0.05$.

Supplementary Table S1
SRM transitions and parameters for RNA samples

Compound	Precursor ion (m/z)	Product ion (m/z)	Cone Voltage (V)	Collision Energy
C	244.24	111.90	16	34
U	245.24	112.90	18	34
A	268.32	135.90	26	38
G	284.33	152.00	18	36
meC	258.24	125.90	16	34
meA	282.32	149.90	26	38

Supplementary Figure S1

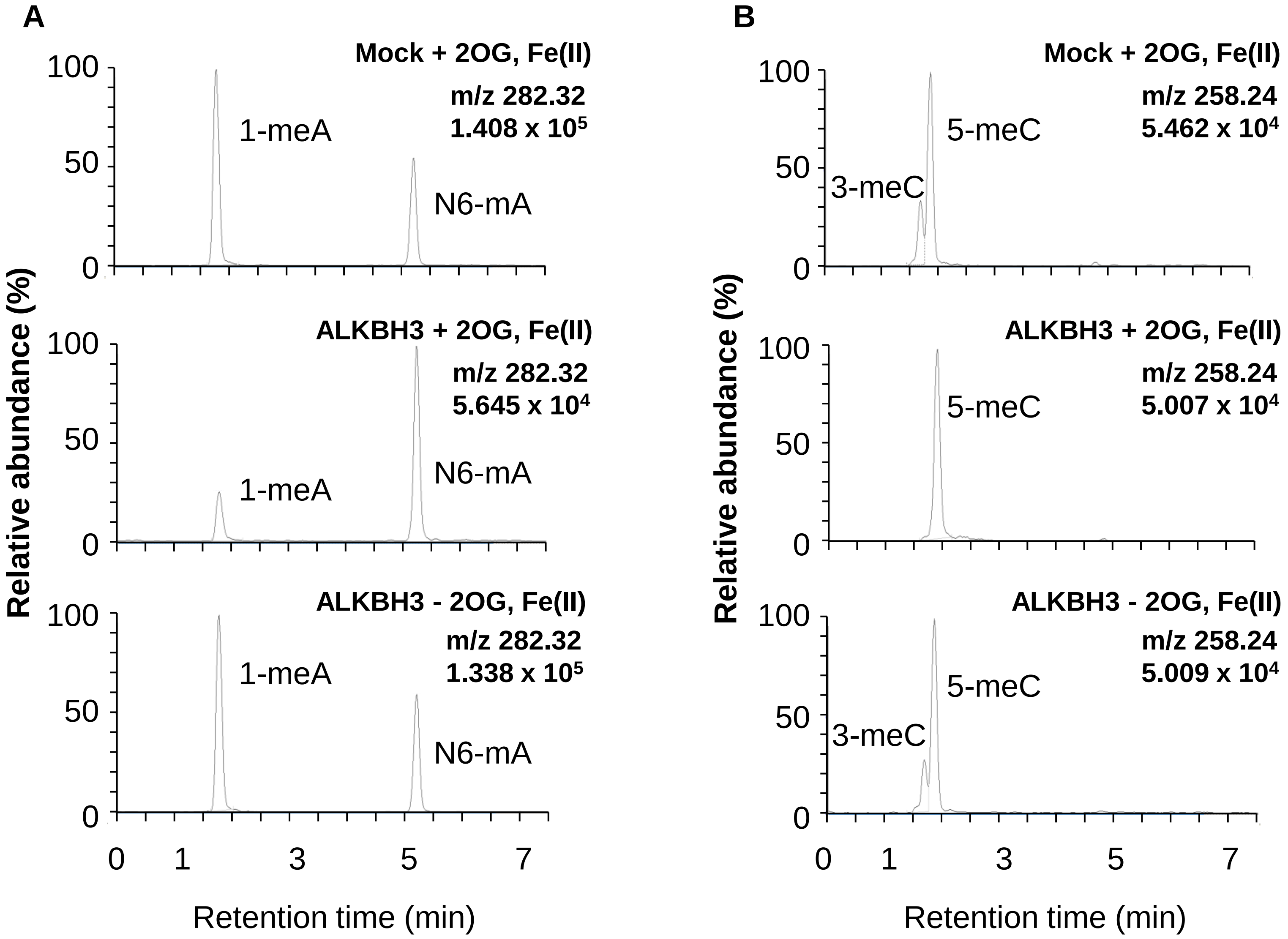

Supplementary Figure S2

A

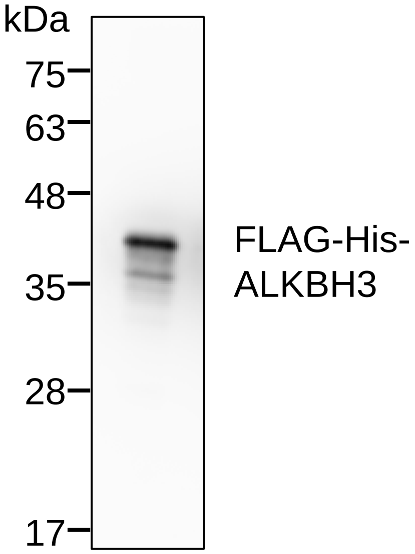

B

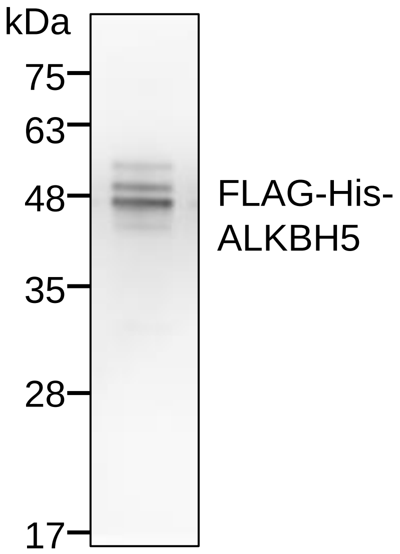

Supplementary Figure S3

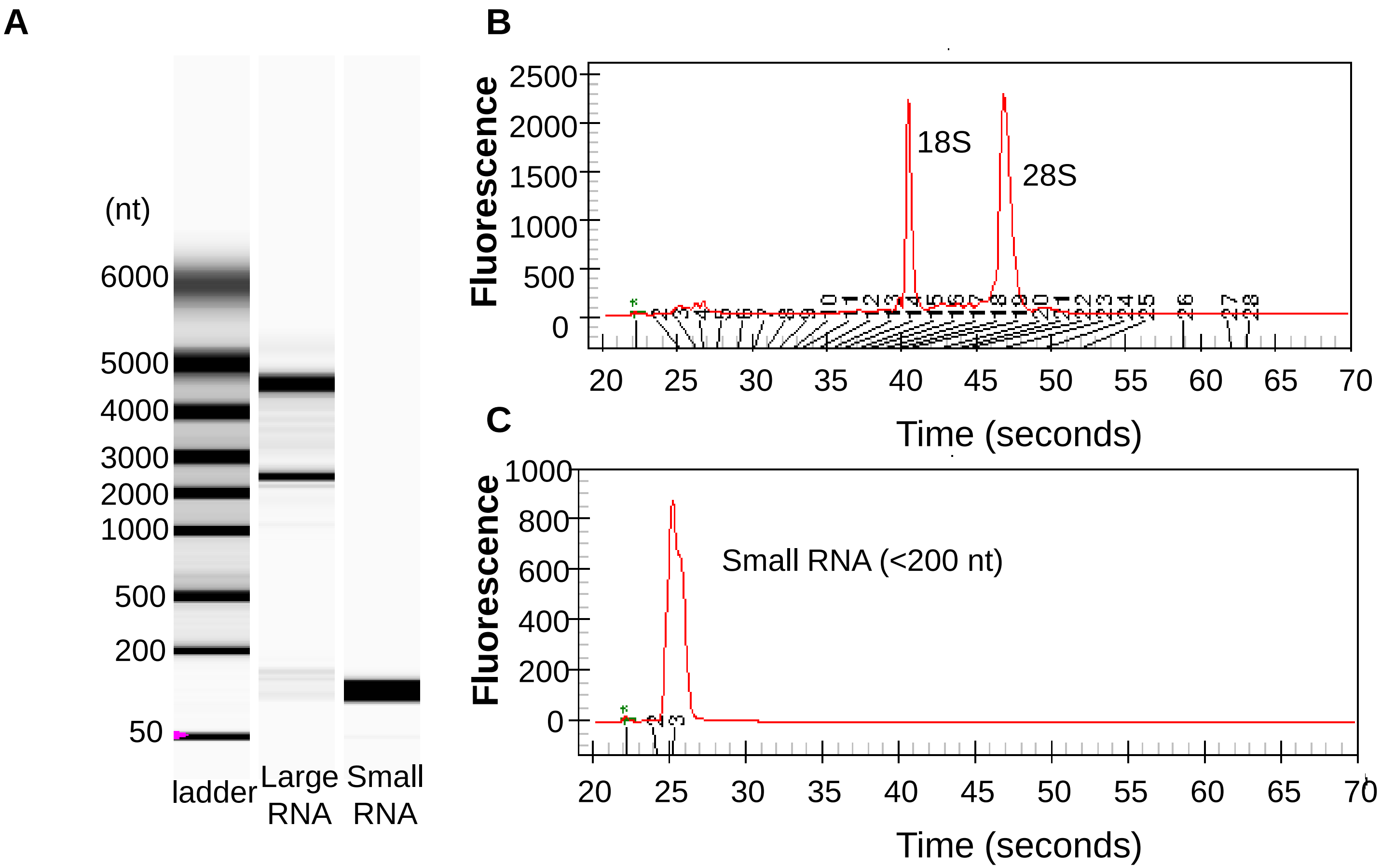

Supplementary Figure S4

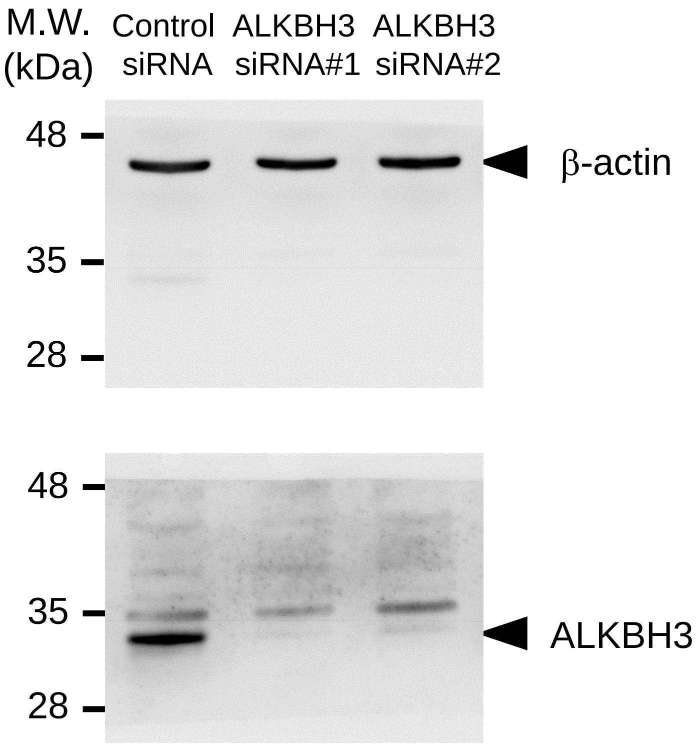

Supplementary Figure S5

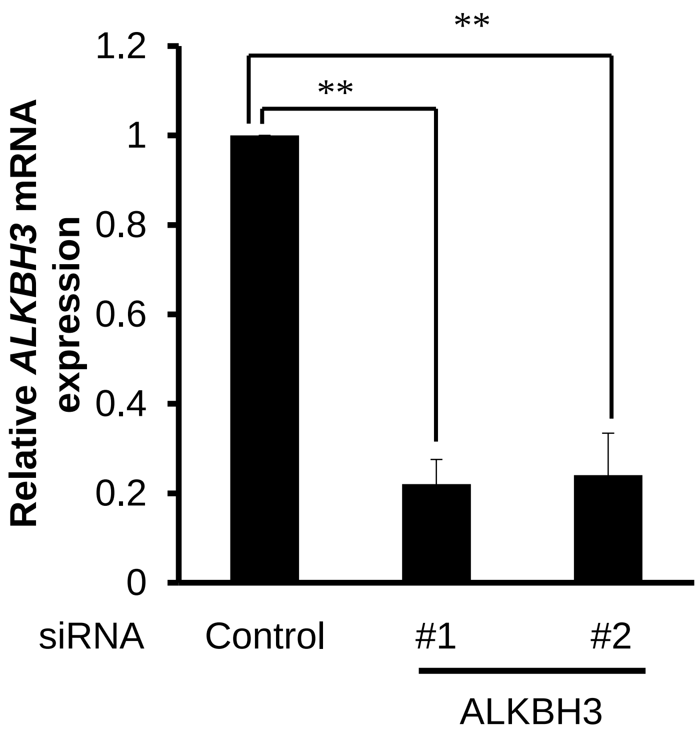

Supplementary Figure S6

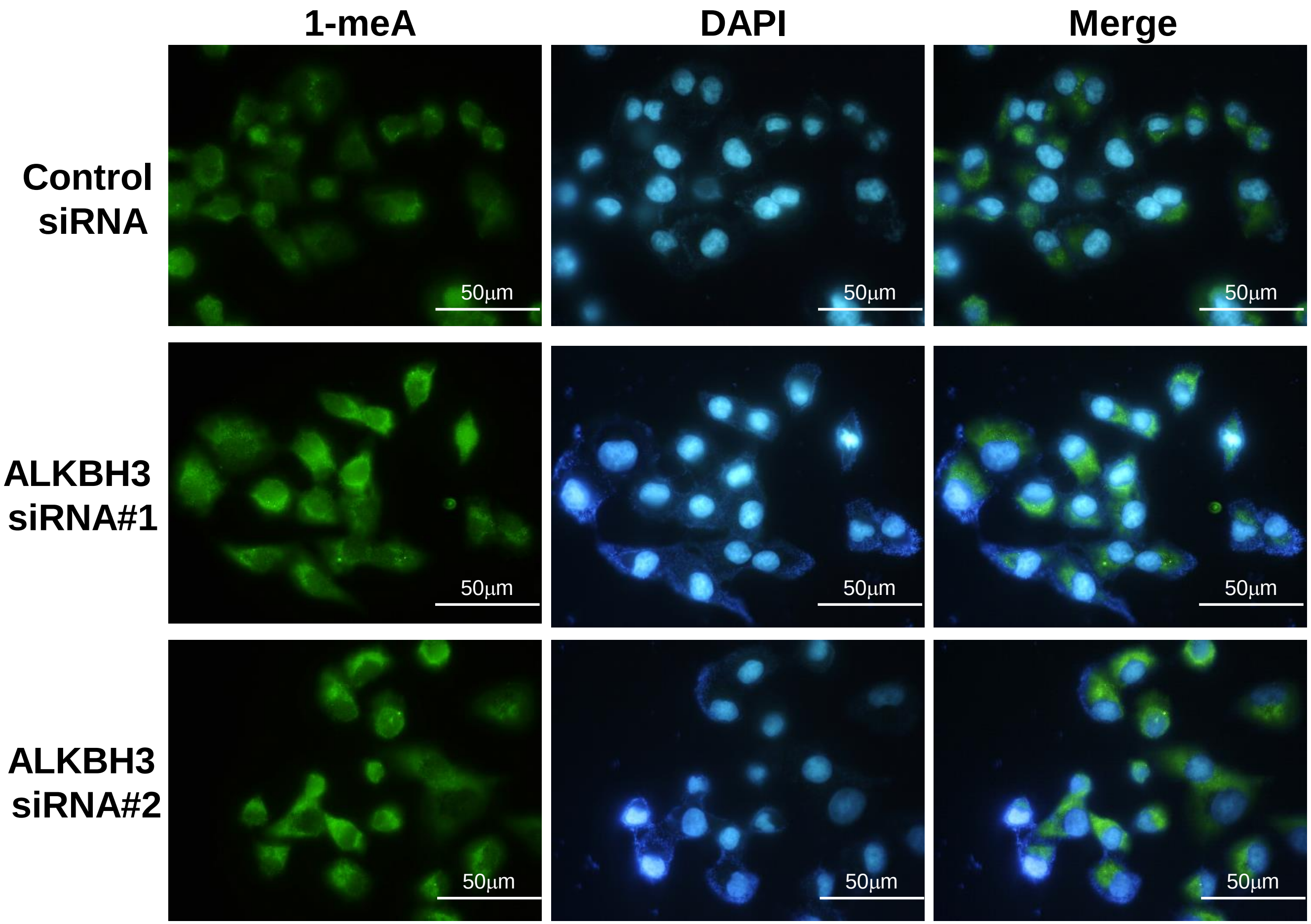

Supplementary Figure S7

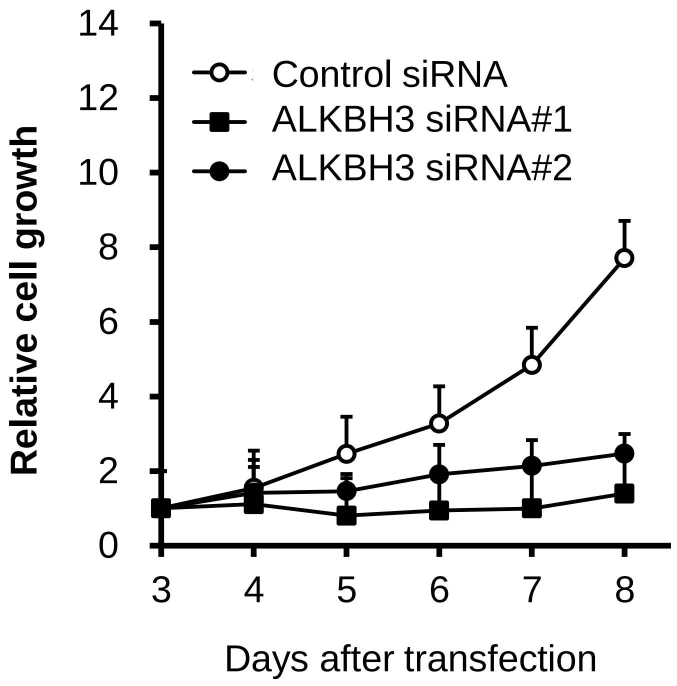

Supplementary Figure S8

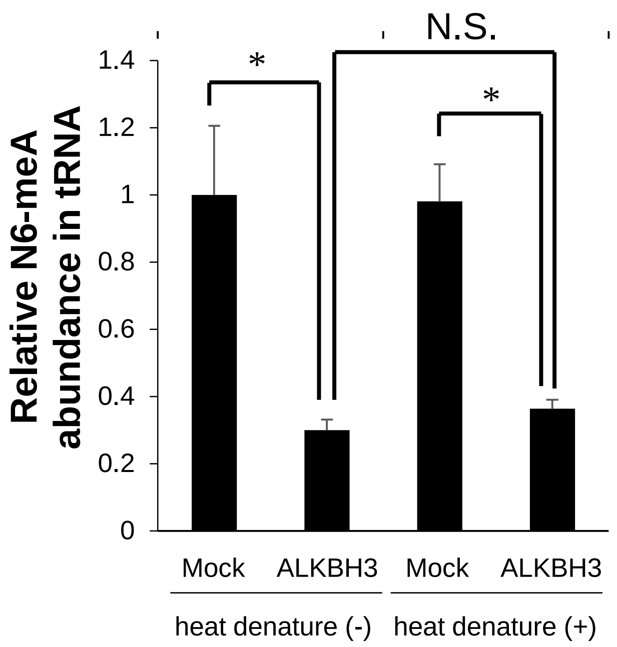

Supplement: Supplementary Information [file srep42271-s1.pdf]
